# Understanding the Political Skills and Behaviours for Leading the Implementation of Health Services Change: A Qualitative Interview Study

**DOI:** 10.34172/ijhpm.2022.6564

**Published:** 2022-03-14

**Authors:** Justin Waring, Simon Bishop, Georgia Black, Jenelle M. Clarke, Mark Exworthy, Naomi J. Fulop, Jean Hartley, Angus Ramsay, Bridget Roe

**Affiliations:** ^1^University of Birmingham, Birmingham, UK.; ^2^University of Nottingham, Nottingham, UK.; ^3^University College London, London, UK.; ^4^Open University, Milton Keynes, UK.

**Keywords:** Organisational Politics, Political Skill, Leadership, Change Management, England, National Health Service

## Abstract

**Background:** The implementation of change in health and care services is often complicated by organisational micro-politics. There are calls for those leading change to develop and utilise political skills and behaviours to understand and mediate such politics, but to date only limited research offers a developed empirical conceptualisation of the political skills and behaviours for leading health services change.

**Methods:** A qualitative interview study was undertaken with 66 healthcare leaders from the English National Health Service (NHS). Participants were sampled on the basis of their variable involvement in leading change processes, taking into account anticipated differences in career stage, leadership level and role, care sector, and professional backgrounds. Interpretative data analysis led to the development of five themes.

**Results:** Participants’ accounts highlighted five overarching sets of political skills and behaviours: personal and inter-personal qualities relating to self-belief, resilience and the ability to adapt to different audiences; strategic thinking relating to the ability to understand the wider and local political landscape from which to develop realistic plans for change; communication skills for engaging and influencing stakeholders, especially for understanding and mediating stakeholders’ competing interests; networks and networking in terms of access to resources, and building connections between stakeholders; and relational tactics for dealing with difficult individuals through more direct forms of negotiation and persuasion.

**Conclusion:** The study offers further empirical insight the existing literature on healthcare organisational politics by describing and conceptualising the political skills and behaviours of implementing health services change.

## Background

**Key Messages**

** Implications for policy makers**

- The implementation of strategic change is often complicated by the local organisational politics of care services.
- Those leading change need to utilise a range of political skills and behaviours to understand and mediate the competing interests of stakeholders.
- Political skills and behaviours are rarely used in a formulaic way but are highly context-specific and dependent on the local patterns of opposition and support for change.
- Health and care leaders could be supported to develop political skills and behaviours.

** Implications for the public**

It can be very frustrating when national or local plans for healthcare reform do not easily or quickly become implemented. One reason for this is because healthcare stakeholders hold different views and agendas about what types of change is needed and how such change happens. These differences lead to forms of political behaviour and conflict. This study shows how health and care leaders can use a particular set of skills and behaviours to understand and cope with the politics of change. These skills can help identify the likely forms of opposition and also offer strategies and techniques for persuading others to engage in change processes.

Health and care services are dynamic ‘political’ arenas.^1^ In this paper, we use the term ‘political’ to indicate the more informal or small ‘p’ politics rather than the more formal or big ‘P’ politics of government or policy-making; accepting that the former is often conditioned by the latter. Such organisational politics is manifest in the micro-level tactics and behaviours that people or groups use to influence the organisation of care in line with their particular preferences and interests.^2^ As such, the micro-politics of healthcare can be seen as stemming from varied and competing interests that people hold about the organisation of care, which often reflect institutionalised ways of working and underlying lines of power.^3-5^ The micro-political processes which stem from these competing interests are often described as operating alongside more formal authority structures in the form of informal lines of power, professional cliques and inter-personal influence.

The micro-politics of healthcare organisations has been repeatedly shown to complicate programmes of change including, for example, the implementation of evidence-based guidelines,^6^ inter-professional teams,^7,8^ quality improvement initiatives,^9-11^ technological innovations,^12^ organisational and management reforms^4,13^ and large-scale system change.^14^ Although the burgeoning implementation science literature acknowledges such contextual influences, these micro-political dynamics are often subsumed within broader organisational factors rather than seen as defining the landscape of change.^8^

There are growing calls for those leading change processes to confront and manage organisational politics more explicitly.^15^ Bate et al^9^ suggest change requires ‘politically credible leaders’ who can broker between competing interest groups and manage political processes. One increasingly prominent idea is that those leading change need political skills to understand and navigate their local political context.^16^ Whilst there is an extensive literature describing the micro-politics of healthcare organisation, especially the potential for professionals to resist change,^4,5,17,18^ much of this stops short of conceptualising the types of political skills and behaviours observed when implementing, negotiating or opposing change. A parallel and more recent body of research describes how political skills can facilitate the implementation of healthcare change, but much of this draws on a particular concept of political skill derived from outside of the healthcare sector.^16^ As such, there is limited empirical research that inductively describes and conceptualisations the political skills and behaviours of implementing change in healthcare services.

This study follows Buchanan’s^19^ view that organisational politics is a socially constructed phenomena and emphasis should be given to the meanings of those directly involved, thereby resulting in a more context-specific understanding from which to better inform future research and leadership development programmes. As such, this research aimed to add to the existing literature on healthcare organisational politics by inductively describing and conceptualising the political skills and behaviours used by those leading change, and to relate these to the existing literature on both healthcare politics and the concept of political skill.

###  Organisational Politics and Political Behaviours

All organisations might be thought of as complex political arenas where varied and competing interests give rise to political turmoil as people try to extert influence in the workplace in line with their particular interests influence in the workplace in line with their particular interests.^15,20,21^ Such micro-politics often comes to the fore during periods of change when competing ‘camps’ form around different agendas.^22^ Management research has, in the past, interpreted such political behaviours as relatively negative, where Machiavellian-like behaviours are driven by self-interest,^19^ but recent research re-interprets political behaviours as potentially more constructive, especially for reconciling competing interests and finding shared solutions.^23^

Healthcare services are rife with organisational micro-politics. As noted above, the growing field of implementation science might not yet fully attend to these ideas, but there is an extensive body of research showing how reforms, service innovations and new ways of working are routinely negotiated, contested and resisted at the micro-level. One prominent line of analysis shows how the managerialisation or corporatisation of Western healthcare has faced opposition from healthcare professionals, especially doctors, who perceive a threat to their clinical autonomy and institutionalised lines of power.^4,5,13,17,25^ For example, Alford’s analysis of US healthcare reforms describes corporate rationalisers as challenging dominant professional interests, explaining such politics in terms of underlying structural interests.^5^ Similarly, Harrison and colleagues’ study of management reforms in the English National Health Service (NHS) shows both how doctors resist policy change, as well as the attempts of managers to persuade doctors, yet these activities are explained in terms of the institutionalised ‘power base’ of doctors to determine matters of diagnosis, treatment and care standards.^4^ More recently, Lozeau et al further describe the strategies used by doctors to corrupt management change by, for example, lobbying senior executives and using expert opinion to challenge management analysis.^24^ Waring and Currie also show how doctors can co-opt change by hoarding performance data, modifying managerial processes and re-stating their technical authority to review conduct.^18^ Currie et al also describe the institutional work involved in maintaining professional power in the face of new service models, highlighting how doctors use their expert knowledge to articulate risks in ways to counter prescriptive change.^17^

Such studies give rich empirical insight into the politics of health service change, especially the behavioural repertoires of doctors to resist change, and to a lesser extent the tactics of managers to counter opposition. They also show the importance of actors’ changing positional power, especially where doctors move into hybrid professional-management positions.^17,18^ However, such studies stop short of developing a distinct conceptualisation of political skills and behaviours, and tend to explain the *micro*-politics of change with reference to wider structural or *macro*-political interests.

In contrast, the strategic management literature has long suggested that those leading change need to develop and utilise a distinct set of political skills and behaviours to understand their local political context, counter local resistance, and find constructive alignment between competing interests.^15,25,26^ The concept of political skill is commonly associated with the work of Ferris and colleagues^27,28^ who define it as ‘[*t]he ability to effectively understand others at work, and to use this understanding to influence others to act in ways that enhance one’s personal and/or organizational objectives.’* They elaborate these skills along four dimensions. Social astuteness describes the ability to read social situations and comprehend the lines of power and influence. Interpersonal influence describes the ability to persuade and convince others, especially through building positive relationships. Networking ability describes the ability to develop and use networks of people, as resources and as alliances of support. Apparent sincerity describes the capacity to display authenticity and integrity to others, even if this is directed at more coercive ends. Significantly, this offers a developed conceptualisation of political skills and behaviours, but one that has been developed largely outside of the healthcare sector.

In their recent systematic review, Clarke et al^16^ show that terms such as political skill, acuity or astuteness have a relatively long history in health professional education, but usually as loose or general characterisations. However, they also find that there has been growing application of the Ferris^28^ conceptualisation in a growing number of health services and implementation studies. For example, Montalvo and Byrne^29^ conclude that nurse leaders’ ‘political skill’ can improve their ability motivate others, ameliorate conflict and improve teamwork. Rogers at al^8^ use the concept to understand the implementation of multi-disciplinary teamwork where managers’ use of political skills can mediate diverse professional interests and creates a shared sense of order around new ways of working. Despite the growing influence of the Ferris conceptualisation, Clarke et al^17^ question whether it promotes a relatively individualistic view of political skills as manifest in the ability of a given person to exert inter-personal influence over others. Moreover, they suggest it lacks empirical grounding in the distinct realities of healthcare organisations. Reviewing the literature, Clarke et al offer a modified framework of political skill comprising ‘personal performance’ including self-belief and resilience; ‘contextual awareness’ of the prevailing political landscape and lines of power; ‘stakeholder engagement’ and ‘networking’ or the ability to connect people in change processes; and ‘influence in formal decision-making’ processes to sustain or legitimise more informal activities.

Summarising the existing literature, one line of research offers rich empirical accounts of the politics of change that tend to explain such politics in terms of structural interest, but stops short of conceptualising of political skills and behaviours.^4,5^ Another line of research more directly analyses the political skills of introducing health services change, but this is guided deductively by a particular conceptualisation of political skill derived from outside the healthcare sector and focuses on relatively generalised leadership skills and capabilities, rather than more context-specific strategies and activities.^8,30^ The study reported in this paper aimed to produce a new inductive description and conceptualisation of the political skills and behaviours used by those leading change in healthcare services.

The purpose of the study requires a number of clarifications. First, the study takes into account Buchanan’s^19^ observation that organisational politics is inherently a socially constructed phenomena that stems from the diverse meanings and values that actors and communities hold about the workplace. As such, this study seeks to understand the local experiences and meanings of healthcare actors as they encounter and reflect upon change processes, from which to develop a new conceptualisation of political skills and behaviours in healthcare settings. This does not deny the salience or relevance of concepts offered by the likes of Ferris et al^28^ but it suggests that qualities other than social astuteness, inter-personal influence, networking ability or apparent sincerity may also be relevant to leading healthcare change. Second, it is important to clarify the relationship between political skills and behaviours. Looking at the strategic management literature, it might be tempting to assume that political skills are used almost exclusively by those leading change when confronting the political behaviours of others. We take the view, however, that all organisational actors are capable of political behaviours and, to a greater or lesser extent, these behaviours are guided by forms of political skill, whether or not they are seeking to promote or resist change. Moreover, it is rarely the enhanced political skills of individual leaders that determines change but rather the interactions between multiple actors engaged in political behaviours. And third, it is important to clarify our view of leadership. Although designated ‘leaders’ clearly have a prominent role in formulating and implementing change, this study focuses on change ‘leadership’ or the idea that change is a process undertaken by many ‘change agents’ working together in a distributed or coordinated way,^31^ albeit with some holding more formal or authoritative positions. As such, the study sought to investigate the reflective experiences and meanings of using, and observing, political skills and behaviours during the processes of implementing change.

## Methods

###  Design

 A qualitative interview study was undertaken that investigated the reported experiences and uses of political skills and behaviours by those leading or participating in the implementation of health services change. The study took a broad and inclusive approach to the definition of healthcare ‘leadership’ and ‘change.’ As outlined above, the study focused on the experiences of leading change as a situated process, rather than a formal role, and where multiple people participate in leading change. Similarly, change was defined broadly, without focusing on a single ‘change agenda,’ to include any intentional process of transforming the organisation of care whether at national, regional or local level, as identified and discussed by participants.

###  Study Setting

The research was carried out with healthcare leaders and other change agents working across the English NHS between 2018 and 2020. The English NHS was created in 1948 as a universal, primarily taxpayer funded, care system inclusive of primary, secondary and specialist care services. The history of the NHS is characterised by periods of reform and reorganisation usually driven by the more formal politics of central government policy-making, but where the implementation of such reforms has often been shaped by the types of organisational politics outlined above.^4,17,18^ Much of the early history of the NHS was characterised by strong government direction with delegated layers of top-down bureaucratic administration, albeit with the medical profession holding significant influence in policy-making and service delivery. From the 1980s to recent times, the NHS has been subject to disaggregation and decentralised as reforms have emphasised the role for markets, competition and patient choice, with consequent risk of fragmentation and lack of integration. During this time, the English NHS has parted company with the approaches taken by the other nations of the United Kingdom, which have tended not to pursue the market approach. As described above, the growth of managerialism and markets as models of service organisation have challenged the perceived status and authority of professions. Since 2015, policies have sought to overcome the problems of fragmentation brought about by competition with greater emphasis on integrated care and collaborative working across the health and social care systems. These structural change in the NHS, and the underlying ideologies they often reflect, set the context for the micro-politics of day-to-day service organisation and delivery and may have implications for the micropolitics that are the subject of the study and manuscript especially as it was conducted at a time (2018-2020) when systems leadership and integrated working are promoted in place of competition.

In line with these shifting modes of governance a number of other noteworthy features of the NHS routinely contextualise the way organisational politics is manifest, including the allocation of financial resources through changing commissioning arrangements, the influence of national targets and other regulatory requirements on strategic planning, the promotion of evidence-based clinical decision-making, evolving expectation around public and patient participation in decision-making, and a growing emphasis of inter-organisational and inter-sectoral integration.^1^ As such, the NHS provides an exemplary site for investigating micropolitics and the political skills and behaviours of implementing change.

###  Sampling and Participants

Taking into account the above, participants were identified and recruited on the basis of being able offer reflective insight into their experiences and uses of political skills and behaviours through their participation in change processes. Sampling aimed to reflect differences across (*i*) career stage (early, middle and late), (*ii*) leadership level and role (team, department, organisation, region, national), (*iii*) care sector (primary, secondary, tertiary, community, mental health, social care), and (*iv*) professional backgrounds (medical, nursing, allied, managerial, etc). In practice, the study team developed a preliminary sampling frame that reflected these criteria, which was populated with potential names based upon the study teams’ pre-existing research and practice networks within the care system. Two immediate issues should be noted. In many, but not all instances, participants were recruited on the basis of their current formal leadership or management role, but the interviews investigated leadership experiences over the courses of their career. Second, the sampling approach created potential for bias through relying on pre-existing contacts, although it also offers enhanced scope for trust and rapport with participants to discuss the potentially sensitive issues of organisational politics. Over 80 people were contacted in writing and 50 agreed to participate in the interview study. Sampling also included recent participants in formal leadership development programmes to understand the extent to which political skills were addressed in such training. Recruitment of this group involved engagement through a national network of NHS management trainees and opportunistic sampling through university-based leadership programmes. This resulted in 8 people agreeing to take part in interview and a single cohort of 8 learners agreed to take part in group interview. On-going reflection on interview data indicated that data saturation was evident at around 50 interviews, and subsequent analysis further suggested a strong degree of inductive saturation. All participants were provided in advance of the interview with a Participant Information Sheet and were asked to give both written and verbal consent at the time of interview.

Of the 66 participants, 37 were female and 29 male; 59 were White British, 4 were Asian or British Asian, and 3 were Black or Black British. In terms of career experience, the sample was categorised into three groups: 10 people with less than 10 years of experience, 23 people between 11-20 years of experience, and 21 people with more than 30 years. It was not possible to determine the career length of 12 participants because the information was not given or they had had non-linear careers. See Table 1 for description summary of participants occupational role and level and Table 2 for organisational type.

**Table 1:** Interview Participant’s Occupational Role/Level

**Role**	
Regional-level director	3
Quality/service improvement	18
External relations/communications	1
Local authority social care management	2
Primary care leadership	1
Medical leadership (hospital/regional)	5
Middle-management (ward, department service)	17
Senior management (executive, board-level)	
Nursing leadership	6
Research leadership	2
Patient/Public	3
Voluntary sector	5
Police leadership (health and care liaison)	1
Non-executive	3
National-level leader	3
National-level service improvement	4
**Total**	**66**

**Table 2:** Interview participants’ organisational affiliation

**Setting/Sector**	
Acute or specialist hospital	30
Primary care	1
Specialist service network	3
Research organisation or university	5
Quality improvement agency	5
Commissioning	3
Ambulance	1
Local authority/social care	3
STP (employed by other organisation)	5 (3 dual roles)
NHS England/improvement	4
Voluntary sector	5
Police service	1
Public representative/organisation	3
Medical trainee	3
**Total**	**66**

Abbreviations: NHS, National Health Service; STP, Sustainability and Transformation Partnership.

###  Data Collection and Analysis

 The qualitative interviews followed a semi-structured topic guide that was designed with the purpose of encouraging participants to reflect upon and recount their experiences and uses of political skills and behaviours, as well as their reflections of how other people use such skills and behaviours when leading change. The topic guide did not include a definition of organisational politics or political skill; rather it was explained to participants that the primary focus was everyday workplace or small ‘p’ politics of change, and they were encouraged to reflect upon and give meaning to this in their own terms. Participants were then invited to ‘tell the story’ of a small number of change processes experienced over their career from which to reflect upon and elaborate their understanding of how organisational politics was manifest and how those involved used political skills and behaviours to implement change. Further questions probed these accounts to understand participants’ views on the political skills and behaviours used by themselves and others. A preliminary topic guide was piloted with seven participants, which led to amendments in the range and structure of questions. All interviews were recorded and transcribed verbatim with written and verbal consent of participants. and participant names were replaced with codes with all data stored on secure university systems.

Interpretative data analysis^32,33^ started with all members of the study team closely reading at least two transcripts to identify apposite descriptions and candidate codes. Three members of the team (JW, SB, JMC) then systematically coded the data, with regular meetings to review interpretation and clarify the consistency of codes. ‘Second-order’ codes were developed through the further categorisation and comparison of codes, which were then aggregated in the form of overarching themes. Thematic analysis centred on identifying the prominent political skills and behaviours used when implementing change, rather than analysing differences within the sample, which led to the production of five distinct dimensions of political skill (see Table 3). In these later stages, themes were related back to the existing literature with the aim of understanding the points of similarity and differences. A secondary aim of the study was to produce more context-relevant evidence on the types and forms of political skill and behaviour that might inform future leadership development.

**Table 3:** Summary of Overarching Themes and Codes

**Theme**	**Empirical Codes**
Personal and inter-personal style	Self-awareness, self-belief, and self-reflection Resilience and perseverance Inter-personal style
Strategic thinking	Understanding the broader political landscape Reading the local political landscape Defining and redefining problems and solutions Understanding what is possible and what is a priority
Communication and engagement	Active listening Asking questions Opening up dialogue Framing strategies Rhetorical strategies and resources
Networks and networking	Identifying and appraising networks Access to personal and professional networks Fostering and mobilising networks Creating alignments Identifying and using key people
Relational tactics with difficult people	Dealing with ‘egos’ Negotiating and dealing with powerful groups Manipulating Engaging with formal organisational structures

## Results

###  Personal and Inter-personal Qualities 

The first theme describes the ‘personal and inter-personal’ qualities described as important for dealing with the politics of change. Most participants described change as protracted, time-consuming, and as emotionally and physically draining. Accordingly, personal resilience and the ability to weather ‘*knock-backs*’ were regarded as important political skills. More experienced participants (ten years plus) talked of ‘*playing the long game*’ and waiting out resistance. Relatedly, many talked about the importance of self-belief and the sense of ‘*doing the right thing*’ when engaging in political behaviour. As well as bolstering personal resolve, this was described as conveying the symbolic significance of commitment and resilience in the face of opposition. Reflecting on their experiences of change, the first illustration highlights the importance of maintaining some emotional distance when facing opposition, whilst the second, describes the importance of having the self-belief of ‘doing the right thing.’

“*I think you have to be resilient as well, you need to not take things personally which is quite a tension really because you’re using a lot of personal skills to sell but then you can’t take any negativity personally” *[WP2-4].

“*I’ve got to live with myself, and I will only do things that I know are right and proper, and that at the end of the day I can put my hand on my heart, and even if something wasn’t quite right, it was done with the best of intent, and it wasn’t a call, to be malicious or anything like that” *[WP2-37].

 Participants also described the importance of self-awareness, especially understanding one’s relative standing or position in the local environment, together with a realistic appraisal of one’s skills and abilities. This was seen important for orienting one’s relationships and developing realistic strategic plans and avoiding the personal risks of being unable to deliver change. Reflecting on the key attributes of political skill, the following participant emphasised the importance of not only cultivating self-awareness but also understanding how others perceive you:

“*So absolutely understand yourself and be challenged about that and therefore understand how others perceive you, because that’s key to being political isn’t it? If you think you’re one thing, but others see you as something else [chuckling], that’s a problem” *[WP2-10].

 More experienced participants described cultivating an appropriate inter-personal style that they enacted with different groups or people, rather than relying on their formal role designation or positional power. Many described their ‘leadership style’ in terms of integrity and authenticity in the belief that this inspires trust and commitment, but which also seemed to reflect a desire not to be regarded as duplicitous or manipulative:

“*There are choices you have to make about how you act in order to try to improve that and make a difference to that in a way that allows you to keep your integrity and trying to do something in the interests of patients and the organisation while recognising that other people are defending their own interests”* [WP2-47].

 In other accounts, dealing with the politics of change involved cultivating a ‘diplomatic’ style where they presented themselves as taking a broader, less parochial view of change, and as seeking peaceful resolution by taking a more neutral or intermediary position. Many also talked of being ‘charismatic’ in their efforts to ‘win-over’ supporters. In contrast, some talked of using, at times, an ‘assertive’ or strong style to face-down opponents and stand-up to dominant groups. What seemed significant, however, was the ability or dexterity to move between such styles based upon an appreciation of the expectations of stakeholders:

“*[Y] ou have to be adaptable. You can’t be the same person, you know, you do say, oh he’s always dry, or he’s always like that. I think the trait for leaders I’ve learned from is that they are adaptable, they... with the juniors they will talk in a different way, to the seniors they will talk in a different way, but not in a condescending way, in any way. You know, when you’re talking at that level, at the junior level, you’re encouraging and supporting”* [WP2-17].

###  Strategic Thinking

 Strategic thinking is a common feature of other leadership and change management constructs, but study participants described two distinct aspects as especially important for organisational politics. The first related to how participants appraised the ‘political context’ of change. Nearly all agreed that change was shaped by the broader contours of the formal policy-making, but more experienced participants talked of a complex interplay between big ‘P’ politics and small ‘p’ politics that involved, not only the ability to appraise the wider policy landscape, but more significantly to understand the relevance to the local setting. As described by one senior nurse leader, strategic thinking was related to their position between the more formal and informal political domains, which was seen as making wider policy change as more relevant to local interests:

“*Be able to look at the wider picture … I did that when I was doing that job to go really wide and bring it down. … so I think you need to have an appreciation of the macro picture”* [WP2-4].

More common was the ability to accurately appraise the local political ‘*landscape*,’ including both the formal lines of authority and informal lines of influence. These informal influences were widely associated with the power of professional networks, the special status of certain departments or teams, or the influential role of key people in the organisation. Again, more experienced participants talked of understanding local political tensions in a historical context and recognising the longstanding and unspoken agendas that permeate the workplace. Reflecting about their involvement in large-scale health system change, the following participant talked about need to understand the plurality of motives and concerns of local actors:

“*The key challenge is to actually understand why people that you interact with are doing what they’re doing and to really try to get to know what their drivers are, what are the things that cause them anxiety every day” *[WP2-43].

 The second set of strategic abilities broadly related to formulating ‘realistic’ strategic plans in the context of the political landscape. Participants talked of the importance of defining and redefining service problems and corresponding solutions in ways that would appeal to local groups. In other ways, participants spoke about the importance of having clarity of purpose coupled with a realistic understanding of what was (or was not) possible given the local political landscape. Importantly, this involved moving from general formulations to specific proposals for change that would be seen as feasible and relevant according to stakeholders’ particular preferences or agendas. Discussing the implementation of the national ‘Sepsis Six’ policy, the following participant illustrates the importance of aligning change with local interests:

“*I’ve always been interested in matching all those things up and looking at the wider picture to then bring it, develop things and bring it through to the frontline. That was how I did things and that always gave it some leverage and validation with whatever level across the organisation, if you hang it on those sort of external drivers”* [WP2-4].

###  Communication and Engagement

The third and most prominent theme related to communication and engagement. This is a well-documented feature of the change management literature,^22^ and so this paper focuses primarily on those activities described as especially relevant to dealing with organisational politics. Almost all described listening as the basis for effective communication and, in particular, understanding stakeholders’ agendas. This was important when trying to read the local political landscape and appraise the ‘sticking points’ that complicated change. In other ways, the practice of listening was described as having an additional performative function for showing empathy with others and giving the impression of being listened to. The following extract illustrates this idea, with reference to the implementation of regional specialist service network involving specialists in multiple hospitals:

“*Oh, absolutely listen, but really, really demonstrate you’re listening. Don’t just pretend and pay lip service to it. If you are asking somebody to give you their view, their advice, their expertise then absolutely listen to them and take notice. Don’t think that you know it all and you can do it all better”* [WP2-3].

 The ‘art of listening’ was linked to the skill of asking the ‘right’ questions in the ‘right’ way. In some situations, asking a particular question could demonstrate an appreciation of a local issue as a way of eliciting support. In other instances, asking naïve questions could help engender dialogue and demonstrate a willingness to learn. Again, the way a question was asked had an intentional and performative effect over and above seeking to acquire insight. More experienced participants talked about the importance of ‘speaking multiple languages’ and varying their communication approach between different audiences, suggesting that adaptability in communication style was important. The following participant, for example, worked in the third sector and described the need to understand the interests of different healthcare organisations:

“*…how can I suss out where I need to influence you? What’s the trigger? Like you said “Oh can I?”, so it’s always [chuckling], it sounds awful. It is always being aware, of the other person and what, therefore, what’s right for their organisation?”* [WP2-37].

 Participants described a range of rhetorical strategies to elicit particular types of responses from stakeholders. These were linked to different ‘resources’ that had appeal to different groups. For example, leaders with a clinical background or managers working in the areas of finance or performance management shared a preference towards using ‘data’ or ‘evidence’ which they would tend to use when seeking to influence others, but equally which others could use to influence them. In contrast, other professional groups, such as nurse leaders or those working as more senior executive leaders tended to value ‘patient stories’ or qualitative accounts that brought to light the ‘human’ perspective of change. These could be mobilised in public forums to set the agenda and need for change. In contrast, some talked of working with ‘experts’ or ‘authority figures’ who had technical knowledge or standing in the organisation and could help justify types of change, often when they themselves had limited standing with a given group:

“*The only thing I can do is be very clear about the numbers… I will develop very, very clear charts and graphs because I think she might understand a picture, and just keep, ‘this is what we’re doing, this is what we’re doing, this is what we’re doing’”* [WP2-4].

 More experienced participants described a range of engagement activities that were intentionally presented as inviting others to ‘set the agenda’ or as giving the impression of ceding authority as a means of enrolling potential opponents in change processes. Although some saw this as a legitimate form of empowerment, for others it seemed a more calculated tactic to reduce opposition by giving others the sense of influence. For example, one senior medical leader recognised the importance of encouraging and enabling others to take control during change processes.

“*I think you probably achieve more if you’re prepared to relinquish your own control and that’s more than just delegation”*[WP2-8].

 In general, participants described their communication activities as intentionally directed towards realising inter-personal or group influence. In other words, communication activities were less concerned with giving or receiving information but with framing change in ways that shaped how others perceive, make sense of, and react to, change.

###  Networks and Networking

 The fourth theme relates to the development and uses of relational networks in the context of implementing change. Participants spoke of these networks in three ways. One view of networks related to their underlying role in articulating and structuring the lines of power in the local political landscape. In this sense, networks were described as groups or cliques in which like-minded people came together in favour of or opposition to given change initiatives. Importantly, participants described methods for identifying and appraising these networks, which overlapped with their communication activities. For example, some discussed asking probing questions to identify key groups, looking at the history of opposition to change, or using informants and other contacts to provide necessary intelligence. With obvious connection to understanding the local political landscape, one hospital board member described the importance of understanding the networks of power and influence in their organisation:

“*I think that understanding relationships, understanding power, understanding interests, understanding stakeholders, those are all political skills and they’re all absolutely crucial to this job” *[WP2-46].

A second understanding of networks was as a source of personal support and resources, when participants saw themselves as being part of and benefiting from social connections. For some, this involved ‘peer networks’ with those in similar leadership roles who could provide reflective support and counsel. Others talked of broader ‘personal networks’ with influential people or trusted colleagues who could provide relevant intelligence about a given group or department. Those participants with established professional backgrounds, eg, doctor, nurses, pharmacists, spoke of their ‘professional networks’ as an important source of support, especially for reinforcing shared values, interests and ident*ities in the face of opposition.*

*“ So if you build up lots of friendships in an organisation and lots of collaborators then you can make things happen. If you don’t life can be very, very hard”* [WP2-29].

“*Clinicians have recognised the benefit of using the network in a political environment to get what they want, or to push through what they want, or to get something they want implemented. In a way that probably is political, isn’t it? It’s the politics of learning that you’ve got this big tool that you can use to your benefit”* [WP2-3].

The third account of networks, or rather networking, was as an active process of creating connections between stakeholders to coordinate, support or implement change processes. Participants described a range of activities that reflected broader perspectives on network management,^34^ and included identifying key people, engendering shared understanding or purpose, coordinating relationships, and sharing resources. However, participants more often focused on the importance of finding ways to bring people together around change processes through finding ‘common ground’ that ensured people would see benefit in working together. Significantly, participants often talked about building alliances as a direct strategy of countering the influence of pre-existing networks and communications. One quality improvement lead described their role as mediating between different people and agendas:

“*So, yeah, I think, 90% of what we do is about brokering conversations between people about trying to find a way to bring people together and to bind them around a common goal and almost being a counsellor between two folk, enabling them to speak”* [WP2-63].

###  Relational Tactics With “Difficult” People 

 In different ways, the above themes represent linked strategies for dealing with organisational politics, but participants also spoke about a distinct set of relational tactics for dealing with ‘difficult’ people, ie, prominent individuals who could use their personal standing to complicate change. It was commonplace for participants to talk about prominent people, figureheads or ‘egos’ who could mobilise opposition to change. These were often senior level actors or high-status professionals, whose standing operated both within and alongside formal authority structures. Describing their involvement in the introduction of a new regional service network, the following participant highlighted the importance of dealing with such opposition:

“*We had to go and smooth some egos. I remember having to go to one of our [clinical] units. There was myself, the medical lead and the chief operating officer of the hospital who met with a senior doctor and thinking that we were meeting with the senior doctor, but when we got there we were met with a barrage of the senior doctor plus his team, I think. There was something around smoothing the ruffled feathers”* [WP2-3].

 Many participants described listening and being responsive to such people, using the techniques described above, but in ways that sometimes appeared to appease or empower these groups. This could include, for example, inviting them to lead a project group or chair a committee. Another participant from a third sector organisation, talked about the challenges of working with healthcare professionals who often assumed a more prominent position. When working with such groups, they outlined the importance of not assuming an overt leadership position and, where possible, allowing other to take the lead:

“*Very much delegating and working to people’s strengths…encouraging others to take the lead ensuring what happens is right rather than who decides what happens. So it’s terribly important that the right thing happens rather than them looking to the boss”* [WP2-38].

Participants talked of ‘*confronting and challenging*’ influential individuals who repeatedly blocked change. Significantly, this was based on a developed appreciation of the prevailing political landscape, especially the ‘red-lines’ or critical issues around a change agenda. These might be associated with top-down policy mandate, regulatory obligations, or performance objectives. Moreover, participants spoke of ‘picking their battles’ and not risking their own status through engaging in unwinnable conflict:

“*[Its] being able to stand up and say ‘I think you’re wrong, and this is why,’ is just as important as you know, being authentic and all of that” *[WP2-1].

 Participants also outlined strategies for negotiating with difficult people. Negotiation tactics appeared to fall into one of two approaches. One was to identify incentives, inducement or ‘deals’ that it was hoped would satisfy the expectations of a resistant group, such as offering additional resources or access to technologies for participating in a new service model. Another approach was to offer ‘compensation’ for the negative consequences of change. In general, these negotiations and skills were described as highly interactive involving ‘offers and counter-offers.’ One participant described the challenges faced in implementing organisational change and the importance of working ‘behind the scenes’ to build relationships and do ‘deals’ in ways that would not compromise the overall change agenda:

“*I think there’s a lot of what you would call back-stage or behind the scenes conversations, so building, firstly trying to build relationships with peers …to say okay this is happening to me, I’m raising it and they would go yeah, I had one of those, but I’ve just tried to deal with that, you would build a coalition” *[WP2-47].

## Discussion

This study aimed to produce a new evidence-base conceptualisation of the political skills and behaviours used by those leading change in healthcare services. The rationale being that the existing research literature provides two relevant but incomplete accounts. On the one hand, there are many rich empirical accounts of the politics of change that tend to explain such politics in terms of structural interests but stop short of conceptualising political skills and behaviours. On the other hand, a growing body of health services research more directly analyses the political skills of change, but this is often guided deductively by a particular conceptualisation of political skill derived from outside the healthcare sector. Following Buchanan’s^19^ view that organisational politics is a socially constructed phenomena that stems from the diverse meanings and values that actors hold about a given workplace, the study reported in this paper investigated the experiences and meanings of political skill and behaviour from the perspectives of those involved in leading health services change from which to inductively develop new descriptive and conceptual understanding.

Before discussing the empirical themes developed through the study, it is worth acknowledging that participants presented a picture of organisational politics that is consistent with the developed literature.^4,13,18^ Through their many and varied accounts, participants reinforced the idea that healthcare services are routinely shaped by micro-political turmoil, especially when influential figureheads, professional cliques or specialist teams mobilise to negotiate or resist change. Nearly all accounts located their experiences of political skills and behaviours in terms of understanding and tackling such opposition, although some described using such skills and behaviours to negotiate planned changed.

Whilst it is often inferred that such *micro*-politics is rooted in the *macro*-political tensions between professional or organisational groups in the form of their structural interests and ideology,^4,5^ only a few participants discussed such deeper interests directly. More often, accounts focused on seeking to maintain the status quo or of apprehension borne out of change fatigue. This is not to say that underlying professional or organisational interests were not at play, but for some participants the political challenge was dealing with a sense of (what they saw as) apathy. More significantly, the study builds on the more critical and structural accounts of healthcare organisational politics in two key ways. The first is through re-focusing analysis back on the experiences and meanings of those directly involved from which to develop a descriptive and conceptual understanding of political skills and behaviours. This does not seek to neglect or downplay the importance of structural interests, but it seeks to re-insert a degree of agency into analysis. The second is through focusing, less on opposition to change, and more on the skills and behaviours of leading change. Too often the literature portrays an image of healthcare politics in terms of ‘doctor versus manager’ or ‘doctor versus nurse’; but this study included participants from multiple professional and organisational backgrounds, where positions of ‘promotion’ and ‘opposition’ were not always aligned to a particular group, but varied over time and according to the nature of the change agenda. In other words, the nature of health service organisational political conflict is not a simple didactic between professional groups, but is varied and complex across many change actors and stakeholders.

Turning to the empirical themes developed through the study, it is important to acknowledge that there are many similarities with the existing health services research literature. That said, the themes offer empirical extensions and clarifications to the existing conceptualisations of political skill. More significantly they provide an understand of political skills and behaviours that is not directly based on Ferris and colleagues’^28^ relatively individualised and generalised view of political skill and, therefore, has the potential to address the specific capabilities and contingencies for the healthcare sector.

The ‘personal and inter-personal qualities’ described in the study have clear similarities with the existing literature, especially Ferris and colleagues’ constructs ‘inter-personal’ and to some extent ‘apparent sincerity.’ However, the findings enrich such generalised constructs by highlighting, first, the emotional implications of healthcare politics and, second, the importance of inter-personal style. Participants described change as emotionally draining, where opposition is directed both at the change agenda and the person leading change. As such, participants highlighted the importance of their personal resilience as a foundation for effective political behaviour. It was also clear that participants described the importance of being adaptable in order to engage different stakeholders, and being able to present different selves according to the interactive context. This moves conceptual thinking beyond the individual level, ie, *Person A* adopts a style to influence *Person B*, to see political behaviours as more interactive and relational, ie, where both styles are inter-dependently constituted through the interaction.

The theme of ‘strategic thinking’ again has close parallels with Ferris and colleagues^28^ construct ‘social astuteness’ and Clarke and colleagues’^16^ ‘contextual awareness’ but it highlights an aspect of political skill that is often neglected in other accounts. Specifically, those leading change in the healthcare sector often need to attend to the dual and linked contextual influences found in both the ‘outer’ context of policy, regulation or financing, and also the ‘inner’ context of overlapping lines of formal authority and informal power between management, professional groups and clinical team. Such broad contextual dimensions are widely acknowledged in implementation research,^35^ but the political dimension can be overlooked.^8^ More significantly, however, is the need for those leading change to find alignments and opportunities between these contextual dynamics.

The theme of ‘communication’ was by far the most prominent, but within the existing literature this is often subsumed within constructs for ‘inter-personal influence,’ ‘networking’ or ‘engagement.’^19,29^ Beyond specific techniques, the findings suggest the micro-politics of healthcare centres on forms of communicative and dialogic inter-action^36^ that operate alongside formal authority structures or isolated instances of interpersonal influence. This is not to say that groups cannot be persuaded through other resources or inducements, but that the ability to persuade through reasoned argument appears to be a significant feature of healthcare organisational politics,^36^ or were at least amongst the most salient for our participants. More significantly, this demonstrates that organisational politics ultimately rests on issues of meaning and value that different groups hold about the organisation of care,^19^ and as such communicating the value of change requires understanding and mediating between the epistemic and pragmatic boundaries that distinguish stakeholders.^37^

The theme of networks and networking is widely recognised within the existing literature. However, the findings suggest that engaging in political behaviour (either to implement or oppose change) goes far beyond the skills to draw upon contacts or make connections, but rather it relies on forms of coordinate and collective action in the form of distributed or shared leadership.^38^ As such, we should think less about *individual* political skills and behaviours and more about *collective* political behaviours, drawn together from the coordinated political skills and behaviours of multiple actors working together in the face of coordinated opposition. The particular forms of political skill and behaviour, both at the individual and collective level, are ultimately manifest in the context of the specific interaction and game-like turn-taking between organisational groups. This is the major point of departure between the findings present in this paper and the concept of political skill that often dominates research.^27,28^

Although inter-personal influence is commonly described in the existing literature, this study found that such influence was often most important when dealing with ‘difficult’ individuals or *prima donnas.*^39^ Furthermore, the skills and behaviours used were often highly context-specific relating to individual responses to change, but also guided by tried-and-tested techniques honed over many years. In other words, influence was less an overall strategy of leaders and more of a targeted tool used in specific contexts. What seems significant, therefore, is the idea that political skills and behaviours are far from formulaic or that they follow a prescribed ‘play book’; rather they are situated responses to the unfolding game-like processes of action, reaction, counter-action.^40^

As suggested above, it is difficult to derive a formulaic model of political skills and behaviours given the interactive complexity and context-specific contingencies. That said, it is reasonable to suggest that some types of behaviours are likely to be more significant at certain stages of a change process. Although forms of self-belief and resilience are important throughout, they are likely to become more important as time goes on and resistance to change sustains. Again, strategic thinking might be expected to be more significant at the preliminary stages of planning and then at critical junctures when resistance becomes pronounced. Communication skills represent a fundamental basis of engagement and influence throughout the life of any change initiative, and what appears significant is the way communication activities contribute to other political behaviours at critical times. For example, the ability to listen to and understand different views is key to strategic thinking, whilst the ability to persuade is essential to the way actors make sense of change and engage in collective action.^41^ Similarly, networking is key to collective action but relies on related capabilities in strategic thinking, communication, and inter-personal influence. The study therefore suggests the thematic components of political behaviour need to combine and complement each other across different stages of the change process.

The study findings have implications for healthcare leadership education and organisational development programmes, especially for enhancing the political awareness, skills and behaviours of future managers and leaders. It is worth acknowledging that prominent leadership programmes within the English NHS already highlight the importance of developing leaders’ political acuity, astuteness and skills,^42^ but these remain relatively general in their characterisation of what this might involve, ie, understanding lines of power and local cultural context. This study can significantly enhance such programmes by detailing the types of skills and behavioural capabilities actually used by healthcare leaders and offering insights into how these can be used to address the politics of implementing change. As such, future training might focus on the particularities of the micro-politics of change experienced by leaders in different contexts and stages of their career through forms of action learning or situated coaching.^43^ There is scope for future research to further develop and test learning materials derived from this study. Beyond thinking about discrete political skills and behaviours, the study findings offer broader understanding of the micro-politics of change that is often missing in leadership development programmes. In particular it highlights the importance, for example, of timing or findings windows of opportunity,^43^ the politics of contextual-adaption and normalisation,and the building of networks or movements.^40^ More detailed apprehension of the politics of implementation might in turn lead into more realistic expectations of the possibilities for and processes of change.

###  Limitations

 The study has a number of limitations that provide opportunities for future research. The first limitation centres on sampling. As described above, sampling relied on identifying and contacting participants through established researcher networks. As such, the majority of participants were known to the study team and, even where they were interviewed by another member of the team, there is the possibility that data would be bias by prior association. That said, a counter-argument is that prior contact with participants could have fostered enhanced rapport and levels of trust, which are regarded as essential for qualitative interviewing, especially when dealing with sensitive issues such as organisational politics. A further sampling issue relates to the range and number of participants, with some occupations or roles, eg, hospital managers, have greater representation than others, eg, third sector representatives. Although this means it is difficult to make particular inferences from those groups with only a small number of representatives, the primary aim of the study was to understand the broader patterns of political skill and behaviour in leading change.

 The second limitation is that the study relies on self-reported perceptions and behaviours, and there is the inevitable risk of hindsight and attribution bias as participants reflect upon on their experiences in ways that distorts their perception of their own or others actions. Relatedly, the self-reported views offer little possibility to compare multiple perspectives on the same phenomena nor is there observational data of how political behaviour is enacted in different contexts.

 Finally, and adding to the above limitation, the interview study did not focus on a specific change agenda, and it was again difficult to understand how leaders at different career stages or in different roles interacted around a common change process. Taken together, these limitations call for future research that might utilise multiple data sources, including observational research, to better understand how the political behaviours of multiple actors come together around a share change agenda. There is also scope for research to investigate how leaders experience organisational politics at different career stages and how this shapes political behaviours.

## Conclusion

Although there is widespread recognition that healthcare services are complex political arenas, there has been little research investigating directly the forms of political skills, strategies and behaviours that leaders and other change agents utilise to understand and manage such politics when implementing change. The study argues that the political skills and behaviours should not be subsumed within other contextual factors known to shape implementation of change,^8^ nor should they be viewed as one of many leadership variables that influence change. Rather political skills and behaviours can be the key defining feature of change in healthcare organisations. As such, it is important to understand what forms these political skills and behaviours take and how those leading change experience and use them. Although different theoretical perspectives on political behaviour and skill exist in the wider social science literature, these typically draw from studies outside healthcare and often emphasise a more individualistic, self-serving and manipulative view of political skill. Focusing on the experiences and meanings of healthcare leaders, this study offers new insight in the types of skills, strategies and behaviours used when implementing change. The practical implications of the study are that implementation research should give more explicit attention to the issue of organisational politics, in general, and political behaviours, in particular, as part of the analysis of successful and problematic change processes. Furthermore, there is scope for more explicit and evidence-based focus on the development of political skills and behaviours in healthcare leadership programmes.

## Ethical issues

 The study was given favourable ethical review by University of Nottingham Ethics Committee (Business School).

## Competing interests

 Authors declare that they have no competing interests.

## Authors’ contributions

 All authors contributed to the design and conduct of the research and writing of the manuscript.

## Funding

 This research was funded by the National Institute for Health Research (NIHR) Health Services and Delivery Research Programme (study reference NIHR HS&DR 16/52/04). The views expressed in this article do not necessarily reflect those of the UK Department of Health and Social Care, English NHS or National Institute for Health Research.

## References

[1] Harrison S, McDonald R. The Politics of Healthcare in Britain. Los Angles: SAGE Publications; 2007.

[2] Buchanan D, Badham R. Power, Politics, and Organizational Change. Los Angles: SAGE Publications; 2020.

[3] Harrison S, Mcdonald R. The Politics of Healthcare in Britain. London: SAGE Publications; 2008.

[4] Harrison S, Hunter DJ, Marnoch G, Pollitt C. Just Managing: Power and Culture in the National Health Service. London: Macmillan; 1992.

[5] Alford R. Health Care Politics. Chicago: University of Chicago Press; 1975.

[6] Harrison S (2002). New labour, modernisation and the medical labour process. J Soc Policy.

[7] Allen D (1997). The nursing-medical boundary: a negotiated order?. Sociol Health Illn.

[8] Rogers L, De Brún A, Birken SA, Davies C, McAuliffe E (2020). The micropolitics of implementation; a qualitative study exploring the impact of power, authority, and influence when implementing change in healthcare teams. BMC Health Serv Res.

[9] Bate P, Mendel P, Robert G. Organizing for Quality: The Improvement Journeys of Leading Hospitals in Europe and the United States. CRC Press; 2007.

[10] Langley A, Denis JL (2011). Beyond evidence: the micropolitics of improvement. BMJ Qual Saf.

[11] Waring J, Allen D, Braithwaite J, Sandall J (2016). Healthcare quality and safety: a review of policy, practice and research. Sociol Health Illn.

[12] Greenhalgh T, Robert G, Macfarlane F, Bate P, Kyriakidou O (2004). Diffusion of innovations in service organizations: systematic review and recommendations. Milbank Q.

[13] Pettigrew A, Ferlie E, McKee L. Shaping Strategic Change. London, England: SAGE Publications; 1992.

[14] Turner S, Ramsay A, Perry C (2016). Lessons for major system change: centralization of stroke services in two metropolitan areas of England. J Health Serv Res Policy.

[15] Pfeffer J. Managing with Power: Politics and Influence in Organizations. Boston: Harvard Business School; 1994.

[16] Clarke JM, Waring J, Bishop S (2021). The contribution of political skill to the implementation of health services change: a systematic review and narrative synthesis. BMC Health Serv Res.

[17] Currie G, Lockett A, Finn R, Martin G, Waring J (2012). Institutional work to maintain professional power: recreating the model of medical professionalism. Organ Stud.

[18] Waring J, Currie G (2009). Managing expert knowledge: organizational challenges and managerial futures for the UK medical profession. Organ Stud.

[19] Buchanan DA (2008). You stab my back, I’ll stab yours: management experience and perceptions of organization political behaviour. Br J Manag.

[20] Mintzberg H. The power game and the players. In: Shafritz JM, Ott JS, Jang YS, eds. Classics of Organizational Theory. London: Cengage Learning; 2015:284-294.

[21] Pettigrew AM. The Politics of Organizational Decision-Making. London: Tavistock; 1973.

[22] Kotter JP, Schlesinger LA (1979). Choosing strategies for change. Harv Bus Rev.

[23] Hartley J, Alford J, Hughes O, Yates S. Leading with Political Astuteness: A Study of Public Managers in Australia, New Zealand and the United Kingdom. London: Australia and New Zealand School of Government; 2013.

[24] Lozeau D, Langley A, Denis JL (2002). The corruption of managerial techniques by organizations. Hum Relat.

[25] Mintzberg H. Power in and Around Organizations. London: Prentice-Hall; 1983.

[26] Clegg S, Carter C, Kornberger M, Schweitzer J. Strategy: Theory and Practice. London: SAGE Publications; 2011.

[27] Perrewé PL, Zellars KL, Rossi AM (2005). Political skill: an antidote in the role overload-strain relationship. J Occup Health Psychol.

[28] Ferris GR, Treadway DC, Perrewé PL, Brouer RL, Douglas C, Lux S (2007). Political skill in organizations. J Manage.

[29] Montalvo W (2015). Political skill and its relevance to nursing: an integrative review. J Nurs Adm.

[30] Montalvo W, Byrne MW (2016). Mentoring nurses in political skill to navigate organizational politics. Nurs Res Pract.

[31] Denis JL, Langley A, Sergi V (2012). Leadership in the plural. AcadManag Ann.

[32] Braun V, Clarke V (2006). Using thematic analysis in psychology. Qual Res Psychol.

[33] Gibbs G. Analysing Qualitative Data. London: SAGE Publications; 2018.

[34] McGuire M (2002). Managing networks: propositions on what managers do and why they do it. Public Adm Rev.

[35] Damschroder LJ, Aron DC, Keith RE, Kirsh SR, Alexander JA, Lowery JC (2009). Fostering implementation of health services research findings into practice: a consolidated framework for advancing implementation science. Implement Sci.

[36] Habermas J. Moral Consciousness and Communicative Action. Cambridge: MIT Press; 1999.

[37] Waring J, Clarke J, Vickers R (2021). A comparative ethnographic study of collective knowledge brokering across the syntactic, semantic and pragmatic knowledge boundaries in applied health research. Evid Policy.

[38] Martin GP, Waring J (2013). Leading from the middle: Constrained realities of clinical leadership in healthcare organizations. Health (London).

[39] Empson L. Leading Professionals: Power, Politics, and Prima Donnas. Oxford: Oxford University Press; 2017.

[40] Strauss A, Schatzman L, Bucher R, Ehrlich D, Sabshin M. The hospital and its negotiated order. In: Freidson E, ed. The Hospital in Modern Society. New York: Free Press; 1963:147-169.

[41] Waring J, Crompton A (2017). A ‘movement for improvement’? a qualitative study of the adoption of social movement strategies in the implementation of a quality improvement campaign. Sociol Health Illn.

[42] National Leadership Centre. NHS Leadership Qualities Framework. London; 2002.

[43] Yates S, Hartley J (2021). Learning to lead with political astuteness. Int Public Manag J.

